# RUNX3 Mediates Suppression of Tumor Growth and Metastasis of Human CCRCC by Regulating Cyclin Related Proteins and TIMP-1

**DOI:** 10.1371/journal.pone.0032961

**Published:** 2012-03-22

**Authors:** Lijie He, Xiaodi Zhao, Hanmin Wang, Peng Zhang, Changcun Guo, Chen Huang, Xiaowei Liu, Fangfang Yao, Yu Chen, Weijuan Lou, Shiren Sun, Daiming Fan

**Affiliations:** 1 Department of Nephrology, Xijing Hospital, Fourth Military Medical University, Xi’an, Shaanxi, China; 2 Xijing Hospital of Digestive Diseases, State Key Laboratory of Cancer Biology, Fourth Military Medical University, Xi’an, Shaanxi, China; Baylor College of Medicine, United States of America

## Abstract

Here we presented that the expression of RUNX3 was significantly decreased in 75 cases of clear cell renal cell carcinoma (CCRCC) tissues (p<0.05). Enforced RUNX3 expression mediated 786-O cells to exhibit inhibition of growth, G1 cell-cycle arrest and metastasis in *vitro*, and to lost tumorigenicity in nude mouse model *in vivo*. RUNX3-induced growth suppression was found partially to regulate various proteins, including inhibition of cyclinD1, cyclinE, cdk2, cdk4 and p-Rb, but increase of p27^Kip1^, Rb and TIMP-1. Therefore, RUNX3 had the function of inhibiting the proliferative and metastatic abilities of CCRCC cells by regulating cyclins and TIMP1.

## Introduction

Renal cell carcinoma (RCC), the relatively uncommon malignant disease with a dismal outcome arising from the adult kidney, afflicts about 150,000 people globally and accounts for nearly 78,000 deaths each year [1]. Clear cell (75–80%), and papillary (10–15%) carcinoma are the two most frequent subtypes of RCC [2]. By now, the etiology and pathogenesis of renal cancer remain unclear, although heterogeneous genetic alterations have been reported, as VHL, which have been found to be inactivated in the 70% to 80% of CCRCC. More great efforts are needed to identify the signature gene, which will provide a conceptual framework for its early detection and effective treatment.

Runt domain family, which contains RUNX1, RUNX2, and RUNX3, are main regulators of gene expression in cell proliferation and differentiation in human. The human RUNX genes encode the α subunit of the Runt-domain transcription factor PEBP2/CBF [3], [4]. Previous report indicated a direct correlation with reduced expression of RUNX3 and development and progression of various human cancers such as in gastric and colon tumor [3], [4], [5], [6]. However, whether and how RUNX3 functions in human CCRCC remains unknown.

In the present study, we investigated the expression and molecular function of RUNX3 in human CCRCC tissues and cells. Our results showed for the first time that restoration of RUNX3 expression directly attenuated the tumorigeneicity and metastasis of CCRCC. Moreover, RUNX3 positively regulates several cell cycle molecules and TIMP-1 of human CCRCC cells.

## Materials and Methods

### Tissue collection and immunohistochemistry

Immunohistochemistry was performed by using the Histostain TM-Plus SP kit [7]. 75 formalin fixed paraffin-embedded specimens of primary CCRCC and the matched adjacent noncancerous tissues were obtained from the Department of pathology in our hospital. Six resected fresh CCRCC and adjacent non-tumorous specimens were collected in the Department of Urology in our hospital and were immediately frozen in liquid nitrogen and kept at 70°C until the extraction of lysate. All samples were obtained from patients who gave informed consent to use excess specimens for research purposes. The use of human tissues in this study was approved by the institutional review board of the Fourth Military Medical University and was done in accordance with international guidelines for the use of human tissues. Immunohistochemistry was done as described using a rabbit polyclonal antibody against human RUNX3 at a dilution of 1∶200 (Active Motif, Carlsbad, CA) [7]. Negative control was performed by replacing the primary antibody with pre-immune mouse serum. All sections were examined and scored independently by two investigators without the knowledge of patients’ outcome.

Expression of RUNX3 was evaluated according to the ratio of positive cells per specimen and staining intensity as described previously [8]. The ratio of positive cells per specimen was evaluated quantitatively and scored as follows: 0 = staining of V 1%; 1 = staining of 2% to 25%; 2 = staining of 26% to 50%; 3 = staining of 51% to 75%; and 4 = staining of > 75% of the cells examined. Intensity was graded as follows: 0 = negative; 1 = weak; 2 = moderate; and 3 = strong. A total score of 0 to 12 was finally calculated and graded as negative (−; score: 0–1), weak (+; score: 2–4), moderate (++; score: 5–8), and strong (+++; score: 9–12).

### Cell culture

Human CCRCC-derived cell lines 786-O, A-498, and human kidney proximal tubular cell lines HKC, HK-2, were cultured with RPMI1640 (HyClone) or DMEM (Gibco) supplemented with 10% fetal-calf-serum, penicillin (100 U/ml), and streptomycin (100 µg/ml), in a CO2 incubator (Forma Scientific) [9], [10].

### Plasmid construction and cell transfection

PSilencer3.1 (Ambion) was used for construction of human RUNX3-siRNA vectors according to manufacturer’s protocol. The specific oligonucleotides RUNX3-F: 5-GATCCTGACGAGAACTACTCCGCTTTCAAGAGAAGCGGAGTAGTTCTCGTCATTTTTTGGAAA-3, RUNX3-2R: 5-AGCTTTTCCAAAAAATGACGAGAACTACTCCGCTTCTCTTGAAAGCGGAGTAGTTCTCGTCA G-3 were annealed and then subcloned into the BamHI/HindIII cloning site of pSilencer3.1 respectively. Full-length human RUNX3 vector (pBK-RUNX3) and the control vector were gifts of professors Prof. Paul J Farrell from Department of Virology, Ludwig Institute for Cancer Research, UK. Cell transfection was performed with Lipofectamine2000 (Invitrogen, Carlsbad, CA) as described in manufacturer’s protocol in our previous work [11], [12]. Briefly, cells were plated and grown to 70–90% confluence without antibiotics and then transfected with 1 µg plasmids. For stable transfecion, G418 (400 µg/ml) was added into cells after 24 h of transfection. For transient transfection, cells were harvested for further experiments after 48 h of transfection. Mixed clones were screened and expanded for an additional 6 weeks. So the transfected cell lines were designated as 786-O-RUNX3, 786-O-Ctrl, HKC-siRUNX3, and HKC-Ctrl respectively.

### RNA extraction and real-time RT-PCR

Real-time RT-PCR was performed to determine the expression levels of target genes RUNX3. Total RNA was extracted from cultured cells using TRIZOL reagent (Invitrogen Life Technologies). cDNA (accession number HGNC:10473 ) was generated by using a TaqMan Reverse Transcription Kit (Applied Biosystems). Real-time PCR analyses were performed with a TaqMan RNA Assay kit (Applied Biosystems). Primer of RUNX3 sequence was designed using Primer Express Software (Version 1.5). The primer-RUNX3 sequence: (Forward) 5′-GACTGTGATGGCAGGCAATGA-3′ and (Reverse) 5′-CGAAGCGAAGGTCGTTGAA-3′. All protocols and data collection were performed on an iCycler (Bio-Rad) according to the manufacturer’s instructions. Each sample was run in triplicate for the target gene and the internal control gene.

### MTT Assay

Cells were cultured on 96-well plates (Corning) at 1×10^4^ cells/well. Viable cells were assayed at 1, 2, 3, 4, 5, and 6 days. Following MTT (Sigma) incubation, the cells were lysed in 150 µl of 100% DMSO and the absorbance was tested at 490 nm using the 96-well plate reader (Dynex Technologies) [13]. Each experiment was performed in triplicate.

### Soft agar assay

Soft agar assay was determined as described previously [7], [14], [15], [16]. Briefly, a volume of 2 ml of 0.5% agar was added to each well of 12-well plate. Cells were detached and mixed with the topagarose suspension at a final concentration of 0.3%, which was then layered onto the 0.5% agarose underlay. The number of foci >100 µm was counted after 17 days. Each experiment was performed in triplicate.

### Cell cycle analysis

Flow cytometry analysis was performed as described [17]. The transfected cells were harversted when they were 70-80% confluent, suspended in 0.5 ml of 70% alcohol and kept at 4°C overnight. The DNA content of stained nuclei was analyzed by a flow cytometer (EPICS XL, Coulter, Miami FL). The cell cycle was analyzed using Multicycle-DNA Cell Cycle Analyzed Software. Each experiment was done in triplicate.

### Cell migration assays

The ability of 786-O-Ctrl and 786-O-RUNX3 cells to migrate was detected using Transwells (8-µm pore size, Corning Costar Corp). The Transwells were put into the 24-well plates. Freshly trypsinized and washed cells were suspended in DMEM containing 1% fetal bovine serum. 5×10^4^ cells/well were placed in the top chamber of each insert (BD Biosciences, NJ), with the non-coated membrane. 500 µl of DMEM containing 10% fetal bovine serum was added into the lower chambers. After incubating for 24 h at 37°C in a 5% CO_2_ humidified incubator, cells were fixed with 95% absolute alcohol and stained with crystal violet. The cells in the inner chamber were removed with a cotton swab and the cells attached to the bottom side of the membrane were counted and imaged under an inverted microscope (Olympus Corp. Tokyo, Japan) at ×200 magnification over ten random fields in each well. Each experiment was performed in triplicate [18].

### Tumorigenicity

Tumorigenicity in Nude Mice was determined as described previously [7], [15]. Two groups of 6 mice each were injected subcutaneously with transfected cells at a single site. Tumor onset was scored visually and by palpitation at the site of injection by two trained laboratory staffs at different times on the same day. Average tumor size was estimated by physical measurement of the excised tumor at the time of sacrifice. Animals were sacrificed 28 days after injection. These tumors were weighed and verified by hematoxylin and eosin (H&E) staining. Nude mice were manipulated and cared for according to NIH Animal Care and Use Committee guidelines in the Experiment Animal Center of the Fourth Military Medical University (Xi’an, Shanxi, Province, P.R. China).

### Western blot analysis

Protein extraction and immunoblot analyses were performed as described [7], [15], [18]. Cell or tissues lysate were respectively separated by SDS-polyacrylamide gel electrophoresis, blotted onto nitrocellulose membrane, and incubated with a primary antibody, including monoclonal antibodies against RUNX3 (Active Motif, Carlsbad, CA), cyclin D1, cyclin E, TIMP-1, TIMP-2, MMP2, MMP9, Tubulin (Santa Cruz Biotech); β-actin (Sigma); cdk2, cdk4, p27^ Kip1^ (BD Biosciences); Rb and pRb (Cell Signaling Technology). After washing, the membrances were incubated with the HRP-conjugated goat anti-mouse or anti-rabbit IgG antibody (Wuhan, Hubei, China) for 2 h at room temperature. Blots were visualized by using the ECL system (Amersham Pharmacia Biotech). Each assay was repeated at least thrice.

### Statistical analysis

The difference between each group was analyzed by Kruskal-Wallis rank test, Student’s test or Mann-Whitney *U* test using Statistical SPSS software package (SPSS Inc, Chicago). Differences were considered statistically different at *P*<0.05.

## Results

### RUNX3 expression was loss or decreased in CCRCC tissues and the derived cell lines

**Figure 1:**
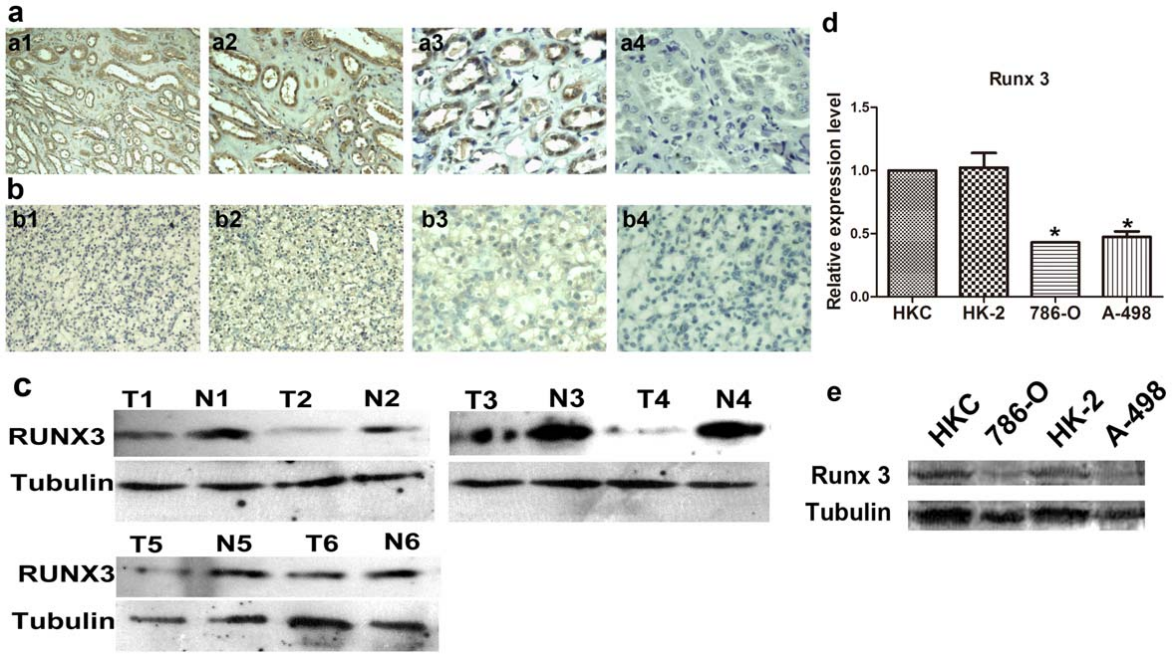
RUNX3 protein expression in CCRCC tissues and the matched noncancerous counterparts. a and b. Representative immunohistochemical photographs were taken at different magnifications in CCRCC tumor tissues and their matched noncancerous counterparts (×100 a1, ×200 a2, ×400 a3 and negative control a4 for noncancerous tissues; ×100 b1, ×200 b2, ×400 b3 and negative control b4 for tumor tissues). c. Expression protein levels of Runx3 in six CCRCC and the matched adjacent noncancerous tissues. d.Expression mRNA levels of RUNX3 in the CCRCC-derived cell lines and human kidney proximal tubular cell lines by real time RT-PCR. 18S was used as an internal control. ^*^ *P* <0.05 vs HKC cells. e. Expression protein levels of RUNX3 in the CCRCC-derived cell lines and human kidney proximal tubular cell lines by Western Blot. Tubulin was used as an internal control.

The expression of RUNX3 was assessed by immunohistochemistry in CCRCC samples with the matched adjacent noncancerous tissues. It was found that RUNX3 was predominantly located in the cytoplasm and nucleus of kidney proximal tubular cells in noncancerous tissues (Fig. 1a). As shown in Table 1, 11 (14.66%), 5 (6.67%), 10 (13.33%) and 49 (65.33%) of the adjacent noncancerous tissues samples had negative, weak, moderate, and strong RUNX3 immunostaining, respectively. On the contrary, 52 (69.33%), 6 (8%), 6 (8%) and 11 (14.67%) of the renal tumor tissues specimens of CCRCC had negative, weak, moderate, and strong RUNX3 immunostaining, respectively (Fig. 1b). The average RUNX3 staining score in adjacent noncancerous tissues of CCRCC was significantly higher than CCRCC (0.853 versus 0.306, *P*<0.05). To provide causal evidence of the role of RUNX3 in CCRCC development and progression, we first determined RUNX3 expression in six human CCRCC tissues and two derived cell lines in mRNA and protein levels, compared with the matched adjacent noncancerous tissues and the human normal kidney proximal tubular cell lines. As shown in Fig. 1c, Runx3 was significantly lower expressed in CCRCC tissues compared with matched adjacent noncancerous tissues. As shown in Fig. 1d and 1e, 786-O and A-498, had a loss or drastic decrease of RUNX3 expression, as compared with their matched the HKC and HK-2 in mRNA and protein levels.

**Table 1:** Clinicopathological associations of RUNX3 expression in patients with CCRCC.

	RUNX3 Expression Level			*P* value
	−	+	++	
Adjacent tissue of CCRCC	12/77(15.58%)	16/77(20.78%)	49/77 (63.64%)	
CCRCC tissues	52/75(69.33%)	12/75(16%)	11/75(14.67%)	<0.001^**^

Note: Interpretation of Runx3 staining was described in Section 2. Runx3 staining was graded as negative (−; score: 0–1), weak (+; score:2–4), and strong (++; score:5–8). Pearson's X^2^ Test, ^**^ *P*<0.001 vs non-cancerous tissues.

### RUNX3 repressed the growth and metastasis of CCRCC cells *in vitro*

**Figure 2:**
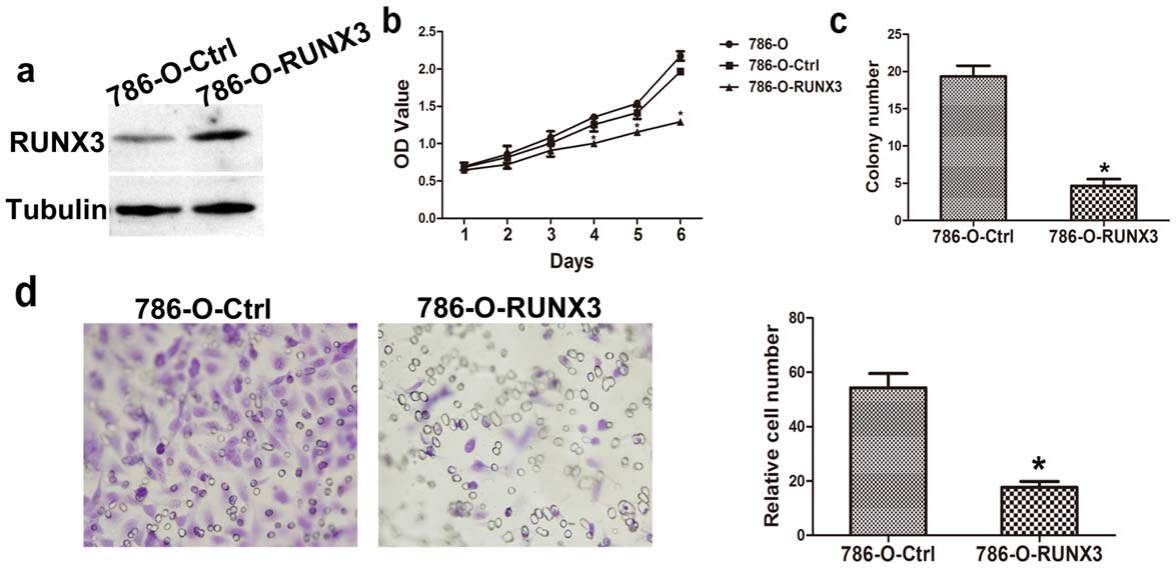
Effects of RUNX3 on the abilities of growth and metastasis in vitro. a. After infection, the expression of RUNX3 in 786-O-Ctrl and 786-O-RUNX3 was evaluated by Western blot. Tubulin was used as an internal control. b. Effect of RUNX3 in regulating 786-O cells proliferation. Monolayer growth rates of 786-O, 786-O-Ctrl and 786-O-RUNX3 cells were determined by MTT assays. Values represent the mean (SEM) from at least three separate experiments. ^*^ *P* <0.05 vs 786-O cells. c. Effect of RUNX3 on colony formation of 786-O cells. Cells were placed in media containing soft agar and incubated for 17 days. The number of foci >100 µm was counted. Values represent the mean (SEM) from at least three separate experiments, each conducted in triplicate. ^*^ *P* <0.05 vs 786-O-Ctrl cells. d. Cell migration assays. Representative fields of migration cells on the membrane (magnification of ×200). Average migration cell number per field. The migration cell number of 786-O-RUNX3 is drastically decreased than that transfected with negative control. ^*^ *P*<0.05 vs 786-O-Ctrl cells, Student’s T-test, n = 10.

PBK-RUNX3 vector was used to up-regulate the expression of RUNX3 in 786-O cells, and the control cells were transfected with the control vector pBK-CMV [11], [12]. After cell transfection and antibiotic screening for 6 weeks, the expression of RUNX3 in stably transfected cells and the control cells pBK-CMV was determined by Western blot. RUNX3 could be significantly up-regulated in 786-O-RUNX3 cells, compared with the control cells 786-O-Ctrl (Fig. 2a). To determine the impact of this restored RUNX3 expression on the growth and metastasis of human CCRCC derived cell lines 786-O, first we determined viable cells and the growth curve using an MTT assay. Restored RUNX3 expression in 786-O cells clearly inhibited the growth of 786-O-RUNX3 cells in vitro (Fig. 2b, P < 0.05), showing that RUNX3 has a tumor-suppressive function in human CCRCC cells. We next studied the ability of these 2 transfected cell lines to form colonies on 6-well cell culture plates for 3 weeks. 786-O-RUNX3 cells have reduced colony formation as low as 2 mM. The number of colonies formed was reduced to 18.6±1.5 compare to 4.8±1.1. Colony formation analysis showed that compared with the control cells, 786-O-RUNX3 transfected cells 786-O-RUNX3 transfected tumor cells have a greatly reduced capacity to form colonies (Fig. 2c, P < 0.05). Because migratory potential is a common feature in the process of tumor metastasis, we then studied the influence of RUNX3 on the migratory abilities of CCRCC cells in vitro by Transwell assay. As shown in Fig. 2d, 786-O-RUNX3 cells and the control ones were through Matrigel on Boyden chamber assay, with average cell numbers of 13.6+4.25 and 55.4±7.62, respectively (*P*<0.05). All these suggested that RUNX3 could inhibit the growth and metastatic abilities of CCRCC cells *in vitro*.

### RUNX3 repressed the tumorigenicity of CCRCC cells *in vivo*

**Figure 3:**
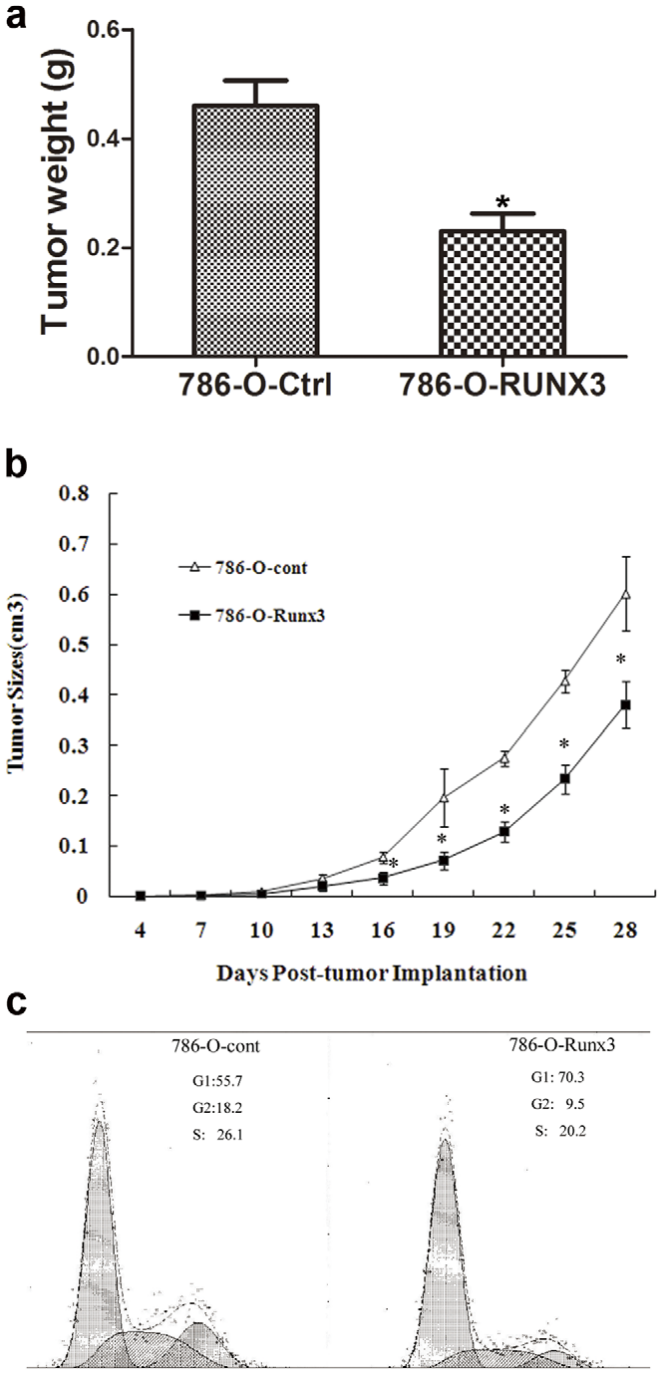
Effect of RUNX3 on tumorigenicity in nude mice and the cell-cycle analysis of 786-O cells. a. Average tumor weight was measurement of the excised tumors at the time of sacrifice. ^*^ *P* <0.05 vs 786-O-Ctrl cells. b. Average tumor size was estimated by physical measurement of the excised tumor at different time. ^*^ *P* <0.05 vs 786-O-Ctrl cells. d. 786-O-Ctrl cells and 786-O-RUNX3 cells were cultured in DMEM for 24 h. Cells were harvested and processed for FACS analysis.

Ectopic expression of RUNX3 protein repressed the tumorigenicity of CCRCC cells by nude mice *in vivo*. As shown in Fig 3a,b, *in vivo* subcutaneous tumor formative assay was adopted to examine the proliferative ability of 786-O-RUNX3 in nude mice. Compared with the control 786-O-Ctrl cells, injection of 786-O-RUNX3 cells led to dramatically decreased tumor weight (Fig. 3a, *P*<0.05) and tumor size (Fig. 3b, *P*<0.05). So the *in vivo* assay suggested that RUNX3 had a potential to inhibit tumorigenicity of CCRCC cells.

### RUNX3 suppressed cell cycle progression of CCRCC cells by targeting cell cycle related molecules

Our data by flow cytometry analysis showed that the cell cycle distribution of 786-O cells was significantly affected by ectopic expression of RUNX3 (Fig. 3c). The cell cycle profile indicates that 70.3% of the 786-O-RUNX3 cells were arrested at G1/S phase, whereas only 55.7% 786-O-Ctrl cells respectively were arrested at the G1/S phase. No significant differences were observed in the fraction of cells in G2 phase. Hence, it indicated that RUNX3 exerted an inhibitory effect on cell cycle progression and this might partly explain the growth suppression effect of RUNX3 on CCRCC cells.

**Figure 4:**
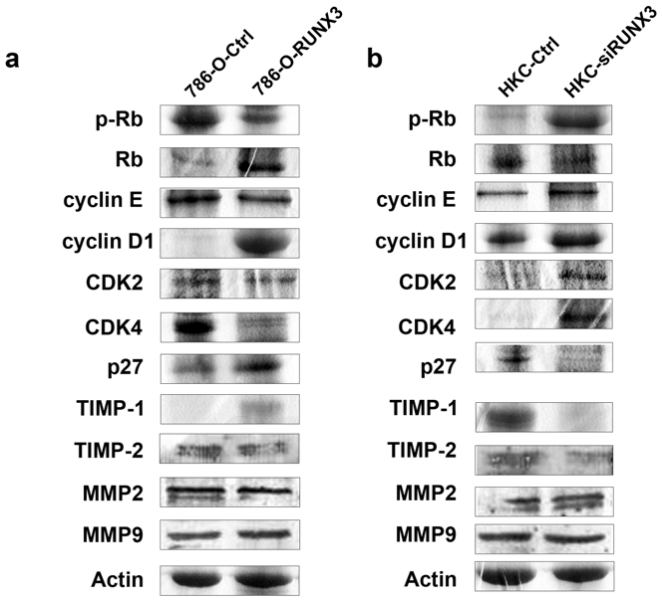
Target genes regulated by RUNX3. a. The expression of cyclin D1, cyclin E, cdk2, cdk4, p-Rb, Rb, p27, MMP2, MMP9, TIMP-1 and TIMP-2 proteins were evaluated in 786-O-Ctrl and 786-O-RUNX3 cells by Western blot. b. The expression of cyclin D1, cyclin E, cdk2, cdk4, p-Rb, Rb, p27, MMP2, MMP9, TIMP-1 and TIMP-2 proteins were evaluated in HKC-Ctrl and HKC-siRUNX3 by Western blot. All examined gene expression levels quantitatively analyzed and expressed as the ratios over β-actin.

In order to explore the underlying molecular mechanism of RUNX3 inducing cell cycle arrest, we detected the expression of cell cycle-related molecules. Our results indicated that ectopic expression of RUNX3 was associated with the reduction of cyclin D1/cdk4, cyclin E/cdk2 and p-Rb but with the increase of p27, Rb protein expression (Fig. 4a). The results were inversed by down-regulating RUNX3 with specific siRNA in HKC cells (Fig. 4b). Therefore, we might infer that RUNX3 induced growth suppression of renal cancer cells partially by regulating various proteins which were controlling G1 to S progression.

### RUNX3 abrogated metastasis of CCRCC by targeting TIMP1

To study the possible role of MMPs in RUNX3-induced inhibition of cell metastasis, ECM degradation is an essential step in tumor invasion and metastasis, which was mainly mediated by the balance between some matrix metalloproteinases (MMPs), such as MMP2 and MMP9 [13], [14] and tissue inhibitors of matrix metalloproteinases (TIMPs), such as TIMP1 and TIMP2 [17], [18]. So we examined the effect of RUNX3 on the expressions of MMP2, MMP9, TIMP1, TIMP2 in CCRCC cells after transfection in 786-O and HKC. Our data confirmed that the expression of TIMP1 could be significantly up-regulated in 786-O-RUNX3 cells (Fig. 4a) and down-regulated in HKC-siRUNX3 cells in protein levels (Fig. 4b). However, the protein expression levels of MMP2, MMP9 and TIMP2 were not changed in 786-O-RUNX3 and HKC-siRUNX3 cells. Taken together, we suggested that inhibiting effect of RUNX3 on metastasis of CCRCC was at least partially mediated by ectopic expression of TIMP-1, which possibly caused the consequent degradation of ECM.

## Discussion

RUNX3, is likely a significant candidate several tumor suppressor gene previously. Our study also provided direct evidence that RUNX3 functions as a tumor suppressor in human renal cancer. Therefore, a better understanding of the molecular basis for the molecular function of the aberrant RUNX3 signaling pathway may help in designing effective therapies to control CCRCC growth and metastasis [19].

In addition, classification into gene ontology categories according to involvement in different biological processes showed that several genes involved in metastasis and cell cycle were changed by RUNX3 [20]. It is interesting to examine the correlation between RUNX3 and these molecules. Because RUNX3 has the potential role in TGF-β signaling, and TGF-β generally induces cell cycle arrest at the G0/G1 phase by increasing the expression or activity of specific cyclin-dependent kinase inhibitors [21], [22], [23] and many other potential targets [24], the tumor suppressor activity of RUNX3 may be realized by inducing cell cycle arrest [25], [26]. Beyond mid-G1, near the end of the G1/S restriction point, the expression of cyclin E with cdk2 has been shown to promote the phosphorylation of Rb [27]. P27 is an important cell cycle inhibitors of the Cyclin D1/cdk4 complexes, cyclin E/cdk2 complexed, exerts its inhibitory activity on many steps of the cell cycle [28]. All these molecules are actually involved in the regulation of cell cycle arrest by RUNX3 is currently under investigation in present work [19]. Our proliferation results demonstrated that RUNX3 might inhibit the renal cancer cell growth by inducing G1/S arrest. Then we tested the expression of cell cycle related molecules in the parent cells and the transfected cells, such as the classic cell cycle regulator Cyclins, cdk and cdk inhibitor molecules. In all, results obtained in the present study provided that RUNX3 exerted its effects on cell cycle progression mainly via inhibiting of Cyclin D1/cdk4 and cyclin E/cdk2 complexes, ectopic expression ofp27^ Kip1^ expression and accelerating of pRb dephosphorylation.

Metastasis is also a mortal factor for cancer patients. By now, the exact metastatic mechanisms of CCRCC are still not fully elucidated. In previous studies, evidence has been accumulated that metastasis of solid tumors require the action of tumor-associated proteases, which promote the dissolution of the surrounding tumor matrix and the basement membrane [29], [30]. As we know, MMPs and their tissue inhibitors, TIMPs played an important role in that process in tumor metastasis [30], [31]. In this study, we observed the relationship of RUNX3 with them. Our study suggested that in CCRCC cells the expression of MMP2, MMP9 and TIMP2 may not be regulated by RUNX3. But RUNX3 could significantly change the expression of TIMP1 levels. All these improved that RUNX3 may be involved in other unidentified mechanisms, such as TIMP1, thereby degrading extracellular matrix and participating in the metastasis of CCRCC.

In all, experimental evidence confirmed that RUNX3 was down-regulated in CCRCC and appeared to be a potent growth and metastasis inhibitor in renal cancer cells. Despite our work suggested that the role of RUNX3 as tumor suppressor might be played through the cell cycle related proteins and TIMP-1, more precise mechanism remained to be elucidated deeply.
